# Supplementary material for: Splicing QTL mapping in stimulated macrophages associates low-usage splice junctions with immune-mediated disease risk
Source: Nat Commun. 2025 Aug 27;16:7205. doi: 10.1038/s41467-025-61669-2 (PMC12391537; doi:10.1038/s41467-025-61669-2)
Supplement: Supplementary file 1 — Supplementary Information [file 41467_2025_61669_MOESM1_ESM.docx]

# Supplementary Note 1

## iPSC culture and macrophage differentiation

iPSC culture and macrophage differentiation was carried as previously described in Alasoo, K. et al.^1^but with some minor modifications.

Feeder-dependent iPSCs were grown on irradiated CF-1 mouse embryonic fibroblast (MEF) feeder cells (AMS Biotechnology) in Advanced DMEM-F12 (Gibco) supplemented with 20% Knock-Out Serum Replacement (KSR) (Gibco), 2mM L-glutamine (Sigma), 50 IU/ml penicillin (Sigma), 50 IU/ml Streptomycin (Sigma) and 50µM β-Mercaptoethanol (Sigma M6250). The media was supplemented with 4 ng/ml recombinant human fibroblast growth factor (rhFGF) basic (R&D, 233-FB-025) to maintain pluripotency and was changed daily. MEFs were seeded on 0.1% gelatine-coated tissue-culture treated plates (Corning 6-well or 10 cm plates) 24 hours prior to passaging iPSCs at a cell density of 2 million cells/6-well or 10-cm plate in Advanced DMEM-F12 supplemented with 10% FBS (labtech), 2mM L-glutamine (Sigma), 50 IU/ml Penicillin and 50 IU/ml Streptomycin (Sigma). Prior to passaging or embryoid body formation, iPSCs were dissociated from the plates using 1:1 mixture of collagenase (1 mg/ml) and dispase (1 mg/ml) (both Gibco).

For EB formation, iPSC colonies were treated with 1:1 mixture of collagenase (1 mg/ml) and dispase (1 mg/ml) and intact colonies were transferred to low-adherence plates (Sterilin). The colonies were cultured in feeder-dependent iPSC medium without rhFGF for 3 days.

For myeloid precursor formation, EBs were harvested 3 days after formation and transferred onto gelatinised tissue-culture treated 10 cm dishes at a rate of 10 EBs per well of a 6 well plate in serum-free X-VIVO 15 (Lonza) or Stem Pro-34 SFM (Thermo Fisher), with both mediums supplemented with 2 mM GlutaMAX (Thermo Fisher), 50 IU/ml penicillin, 50 IU/ml streptomycin (Sigma), 100 ng/ml human macrophage colony stimulating factor (hM-CSF) (Peprotech) and 25 ng/ml human interleukin-3 (hIL-3) (Peprotech), changing the medium every five days. Depending on the iPSC line, myeloid precursor cells become visible as round floating cells at around 10 to 15 days in culture.

For myeloid to macrophage differentiation, myeloid progenitor cells in suspension were harvested and filtered through a 40 mm nylon filter before centrifuging at 290 g for 3 minutes before resuspending in macrophage complete media (RPMI 1640 (Thermo Fisher) supplemented with 10% heat-inactivated FBS (Thermo Fisher), 2mM GlutaMAX (Thermo Fisher) and 100 ng/ml hM-CSF (Peprotech)) at 75 cells/mL. Cells were plated at a density of 10,000 cells per well on a 96-well plate (for RNA-seq), or 25,000 cells per well of black 96-well plate (VWR) (for the macrophage purity assay) and differentiated for another 7 days.

## iPSC-derived macrophage purity assay

iPSC-derived macrophages progenitor cells were seeded and differentiated as above before fixing in 50 µL of 4 % formaldehyde (Applichem, A0823.2500) at 4°C for 20 minutes. Cells were washed twice in 100 µL PBS with calcium and magnesium (Sigma, D8662) before blocking in 10% (v/v) donkey serum (AbD Serotec, C06SBZ) 0.1% Triton X-100 (Sigma, 93420) at room temperature for 1 hour. Cells were then stained with 1:200 anti-CD14 (BioLegend, 301802) and 1:800 anti-CD68 (Cell Signalling Technology 76437S) in 1% blocking solution overnight at 4C. We then washed the cells three times with PBS and stained with secondary antibodies (1:1000 donkey anti-mouse AF647 and 1:1000 donkey anti-rabbit AF488) and DAPI (10ug/mL, AppliChem A1001) at room temperature for 1 hour. Wells without primary antibody were used as negative staining controls. Cells were washed three times and imaged on a Cellomics Arrayscan (ThermoFisher), and the proportion of CD14+CD68+ cells calculated. Only cell lines with greater than 90% double stained cells were processed for RNAseq.

## iPSC-derived macrophage stimulation conditions

After the 7-day differentiation to macrophages, cells were incubated for 6 or 24 hours in complete macrophage media on its own (controls) or containing the following stimuli: : 10 ng/mL recombinant human interleukin-10 (Peprotech, 200-10-2), 10 ng/mL recombinant human interferon-b (Peprotech, 300-02BC-5), 20 ng/mL recombinant human interleukin-4 (Peprotech, 200-04-5), 50 ng/mL P3C (Pam3CSK4) (Tocris, 4633/1), 20 ng/mL recombinant human interferon-γ (Peprotech, 300-02-20), 10 ng/mL lipopolysaccharides from Escherichia coli O127:B8 (Sigma Aldrich, L3129), 40 ng/mL human recombinant tumour necrosis factor alpha (Peprotech, 300-01A-10), 100 ng/mL R848 (Resiquimod) (Invivogen, tlrl-r848), or 5 ng/mL recombinant human sCD40 Ligand (Peprotech, 310-02-10). For the stimulations with HMW poly I:C (Invivogen, tlrl-pic), macrophages were transfected with poly I:C as follows: 0.15 mL of 1 mg/mL of poly I:C was mixed with 0.3 mL P3000 reagent (Lipofectamine 3000 kit) and 5 mL Opti-MEM (Thermo Fisher, 31985062). In another tube, 0.3 mL of Lipofectamine 3000 (Thermo Fisher, L3000001) and 5 mL of Opti-MEM were mixed. The diluted Lipofectamine 3000 and poly I:C tubes were mixed and incubated at room temperature for 10 minutes to allow complexes to form. 100 mL of macrophage differentiation media from above was added to the poly I:C complexes and mixed. Media was removed from the macrophages and replaced with the diluted poly I:C complexes. Control transfections were carried out in exactly the same way but without the addition of poly I:C.

## iPSC-derived macrophage low-input bulk RNA-seq preparation

At the end of the macrophage stimulation period, the media was removed and cells were lysed immediately by adding 50 µL of a 1x lysis/binding buffer (100 mM Tris-HCl pH 7.5, 0.5 M LiCl, 10 mM EDTA, 1 % w/v lithium dodecyl sulphate, and 5 mM 1,4-dithiothreitol) and mixed well. Lysed cells were stored at -80 °C until needed. Using the automated Zephyr G3 NGS Workstation (Perkin Elmer), mRNA was purified from the cell lysates in 96 well plates using the mRNA DIRECT kit (Thermo Fisher, 61012), according to the manufacturer’s instructions, using 20 µL of oligo dT Dynabeads. The purified mRNA was eluted in either 7 µL of nuclease-free 10 mM Tris-HCl pH 7.5 for processing through the modified Smart-seq2 method, or 5 mL of nuclease-free water for processing through the NEBnext Ultra II Directional RNA Library kit (E7760L). For the modified Smart-seq2 method [^65^](https://paperpile.com/c/3m9ohG/ciYt6)), the purified mRNA was processed as follows​​ : 2 µL of oligo dT_30_VN (Integrated DNA Technologies) and 2.34 µL of 10 mM dNTPs (Thermo Fisher, R0193) were mixed with 7 µL of the purified mRNA and heated to 72 °C for 3 minutes to denature secondary structures, before rapidly chilling on ice for 5 minutes. 5 µL of 5x SMARTScribe first-strand buffer (Clontech Takara, 639538), 0.63 µL of SUPERase inhibitor (Thermo Fisher, AM2696), 1.25 µL of 100 mM 1,4-dithiothreitol, 5 µL of betaine (Sigma, B0300-5VL), 0.15 µL of 1 M MgCl_2_, 0.38 µL of template-switching LNA-oligo (TSO) (Qiagen) and 1.25 µL of SMARTScribe reverse transcriptase (Clontech Takara, 639538) were added to the denatured mRNA/dNTP/oligo dT_30_VN mix. Following a brief vortex mix, reverse transcription was performed at 42 °C for 90 minutes, followed by 10 cycles of 50 °C for 2 minutes, then 42 °C for 2 minutes. The reaction was stopped by incubating at 70 °C for 15 minutes. The first-strand cDNA was purified using 0.8 volumes of Ampure XP beads (Beckman Coulter, BCAG0006) to 1 volume of the reverse transcription reaction volume, according to the manufacturer’s instructions, but leaving the eluted cDNA in 12 µL of 10 mM Tris-HCl pH7.5 with the beads in solution. This was done to maximise the amount of cDNA carried forward to the subsequent cDNA amplification reaction. The cDNA was amplified by adding 0.5 µL of 10 µM ISPCR primer (Integrated DNA Technologies) and 12.5 µL of 2x KAPA HiFi polymerase (Kapa Biosystems, KK2601) to the 12 µL of cDNA and mixed before heating at 98 °C for 3 minutes, followed by 11 cycles of 98 °C for 20 seconds, 67 °C for 15 seconds and 72 °C for 6 minutes, followed by a final extension at 72 °C 5 minutes. The amplified double-stranded cDNA was purified as before, but this time the Ampure XP beads were removed from the 20 µL eluate. Amplified double-stranded cDNA was quantified with a Quant-iT^TM^ dsDNA high sensitivity assay kit (Thermo Fisher, Q33120) in black v-bottom 96-well plates (Greiner Bio-One, 651209) on a FLUOstar Omega (BMG Labtech), according manufacturers’ instructions. For cDNA tagmentation, 4 ng of cDNA was diluted with 10 mM Tris-HCl pH 7.5 to a volume of 9.5 µL. 5 µL of a 3x tagmentation buffer (99 mM Tris acetate, 198 mM potassium acetate, 30 mM magnesium acetate and 48 % v/v N,N-dimethylformamide) and 0.5 µL of TDE1 (Illumina, 20034197) were added, mixed and incubated at 55 °C for 5 minutes. The tagmentation reaction was stopped by the addition of 2.5 µL of a tagmentation stop buffer (220 mM EDTA and 1.1 % w/v sodium dodecyl sulphate) and mixed before incubating at room temperature for 10 minutes. The tagmented cDNA was diluted with 10 mM Tris-HCl pH 7.5 to a final volume of 50 µL, before purifying with a 2:1 ratio of Ampure XP beads to sample volume, eluting the tagmented cDNA in 7 µL of 10 mM Tris-HCl pH 7.5. Tagmented cDNA samples were then amplified and sample-indexed by PCR as follows: 7 µL of tagmented cDNA was added to 2.5 µL of i5 index adapter and 2.5 µL of i7 index adapter from the Nextera® XT index kit v2 set A (Illumina, 15052163), 0.25 µL of 50 µM PC1 primer, 0.25 µL of 50 µM PC2 primer and 12.5 µL of 2x KAPA HiFi polymerase, before mixing and incubating at 72 °C for 3 minutes, 98 °C for 30 seconds, followed by 9 cycles at 98 °C for 15 seconds, 62 °C for 30 seconds and 72 °C for 30 seconds, followed by a final extension at 72 °C for 3 minutes. Individual libraries were purified, excess primers removed by performing 0.8:1 ratio of Ampure XP beads to PCR volume, eluting the finished library in 20 µL of 10 mM Tris-HCl pH 7.5.mRNA processed through the NEBnext Ultra II Directional library kit was done so according to the manufacturer’s instructions with 17 cycles of PCR.

All libraries were quantified with a Quant-iT^TM^ dsDNA high sensitivity assay kit, as mentioned above, before combining 96 libraries per pool in equimolar amounts. Library pools were assessed for fragment length and quantity on a Bioanalyser using a High Sensitivity DNA kit (Agilent Technologies, 5067-4626), according to the manufacturer’s instructions. Each 96-library pool was sequenced over 8 lanes of a HiSeq SBS v4, collecting 75 bp paired-end reads.

[1] Alasoo, K., Rodrigues, J., Mukhopadhyay, S. et al. Shared genetic effects on chromatin and gene expression indicate a role for enhancer priming in immune response. Nat Genet 50, 424–431 (2018). https://doi.org/10.1038/s41588-018-0046-7

Supplementary Note 2

We performed pathway enrichment analysis using the REACTOME 2016 database, where biological pathways are organised in a hierarchical structure. Each broad high-level pathway includes lower-level pathways with increasingly specific pathway categories. Although we found differences in pathway enrichment between different conditions and timepoints at low-level pathways (e.g. RIG-I/MDA5 pathway in macrophages stimulated with viral mimic PolyI:C but not in macrophages stimulated with bacterial stimulant sLPS), we noted that a relatively small number of conditions faithfully capture all the high-level enriched pathways (level-2 pathways).

For conditions where RNA is harvested six hours following stimulation, macrophages stimulated with sLPS, either alone or with other stimulants (i.e. LPS+IL10 or LIL10 and CD40+IFNG+LPS or CIL) capture all the level-2 pathways enriched across all 6-hour  conditions (Supplementary Figure ). Almost none of the other 6-hour conditions have any enriched level-2 pathways, suggesting that stimulation with LPS captures most of the pathways that are subject to differential splicing six hours following macrophage stimulation. This is consistent with a rapid response that macrophages exhibit towards activation with bacterial stimulants. On the other hand, viral stimulants and anti-viral cytokines tend to elicit a late response. Stimulation with PolyI:C and IFN β captures the same set of differentially spliced pathways after 24 hours (Supplementary Figure 29). It is expected that PolyI:C and IFNB have similar effects as PIC is known to lead to a strong but delayed production of IFN β and Interferon-stimulated genes (ISGs)^1^.

It has been previously shown that while the macrophage response to LPS and PIC/IFN β implicates overlapping pathways, response to PolyI:C and IFN β is not immediate, while response to LPS starts within a few hours and subsides after 12 hours. We therefore conclude that stimulation with LPS and PolyI:C or IFN β capture most differentially spliced pathways but at different timescales.

In this Supplementary Note, we now highlight some of the key high-level pathways that are enriched in differentially spliced genes:

**Cytokine Signalling and Trafficking Pathways**

Cytokine signalling pathways included pro-inflammatory cytokine pathways such as Toll-like receptor signalling pathways, interferon signalling pathways, signalling by interleukins. LPS binds to TLR-4 which initiates the MyD88-dependent signalling cascade that leads to the production of pro-inflammatory cytokines. Additionally, a TRIF-dependent signalling pathway leads to the production of Type I interferons (reviewed in ref. 2). Similarly, PIC is a potent activator of TLR-3 leading to a TRIF-dependent activation of Type I Interferons. Our enriched pathways recapitulate the interferon and interleukin signalling pathways (Figure) ^3-5^. Additionally, membrane trafficking pathways were enriched for differentially spliced genes in the same conditions. Membrane trafficking is crucial for a variety of functions including trafficking pathogens and pro-inflammatory cytokines produced in response to stimulation. In macrophages, membrane trafficking is a central component of cytokine production. As macrophages lack secretory granules, which in other cell types are used to store cytokines for rapid release^6^, macrophages rely on membrane trafficking pathways to export cytokines as they are released^7,8^.

**Apoptosis**

Pathways related to apoptosis and cellular death were also enriched in differentially spliced genes. Apoptosis plays central roles in pathological processes such as atherosclerotic plaque formation. In the early stage of plaque formation, classically-activated M1 macrophages produce inflammatory cytokines that lead to increased inflammation in response to the accumulation of lipoproteins. In later stages, macrophage apoptosis plays an important role to limit the accumulation of inflammatory cytokines produced by macrophages (reviewed in [9]).

**Splicing auto-regulation**

Interestingly, within the broad pathway “Processing of Capped Intron-Containing Pre-mRNA”, the “mRNA splicing pathway” was significantly enriched in sLPS_6, CIL_6, PIC_24, IFNB_24. Differentially spliced genes include genes essential for the splicing of nascent mRNA such as heterogenous nuclear ribonucleoproteins (hnRNPs L, M, K, UL1, F, A2B1, D, and C), and splicing factors (SRSF11, SRSF5 and SRSF3 and SF3B1). It has been previously shown that the overall levels of splicing regulators are often auto-regulated by adjusting their own splicing to increase the production of unproductive isoforms (splicing auto-regulation; reviewed in [10]). These results suggest that this mechanism may also be engaged upon macrophage exposure to pathogens such as LPS and PIC and inflammatory cytokines such as IFNB.

### References

[1] Reimer, Thornik et al. “poly(I:C) and LPS induce distinct IRF3 and NF-kappaB signaling during type-I IFN and TNF responses in human macrophages.” Journal of leukocyte biology vol. 83,5 (2008): 1249-57. doi:10.1189/jlb.0607412

[2] El-Zayat, S.R., Sibaii, H. & Mannaa, F.A. Toll-like receptors activation, signaling, and targeting: an overview. *Bull Natl Res Cent* **43**, 187 (2019). https://doi.org/10.1186/s42269-019-0227-2

[3] Komal, Asma et al. “TLR3 agonists: RGC100, ARNAX, and poly-IC: a comparative review.” *Immunologic research* vol. 69,4 (2021): 312-322. doi:10.1007/s12026-021-09203-6

[4] Lim, Chan Seok et al. “TLR3 forms a highly organized cluster when bound to a poly(I:C) RNA ligand.” *Nature communications* vol. 13,1 6876. 12 Nov. 2022, doi:10.1038/s41467-022-34602-0

[5] Alexopoulou, L et al. “Recognition of double-stranded RNA and activation of NF-kappaB by Toll-like receptor 3.” *Nature* vol. 413,6857 (2001): 732-8. doi:10.1038/35099560

[6] Marks, Michael S et al. “Lysosome-related organelles: unusual compartments become mainstream.” *Current opinion in cell biology* vol. 25,4 (2013): 495-505. doi:10.1016/j.ceb.2013.04.008

[7] Manderson, Anthony P et al. “Subcompartments of the macrophage recycling endosome direct the differential secretion of IL-6 and TNFalpha.” *The Journal of cell biology* vol. 178,1 (2007): 57-69. doi:10.1083/jcb.200612131

[8] Murray, Rachael Z et al. “Syntaxin 6 and Vti1b form a novel SNARE complex, which is up-regulated in activated macrophages to facilitate exocytosis of tumor necrosis Factor-alpha.” *The Journal of biological chemistry* vol. 280,11 (2005): 10478-83. doi:10.1074/jbc.M414420200

[9] Seimon, Tracie, and Ira Tabas. “Mechanisms and consequences of macrophage apoptosis in atherosclerosis.” *Journal of lipid research* vol. 50 Suppl,Suppl (2009): S382-7. doi:10.1194/jlr.R800032-JLR200

[10] Ding, Fangyuan et al. “Dynamics and functional roles of splicing factor autoregulation.” *Cell reports* vol. 39,12 (2022): 110985. doi:10.1016/j.celrep.2022.110985

## Supplementary Figures

Supplementary Figure 1: Pairwise intron-usage correlation between conditions.

Heatmap and dendrogram showing pairwise Pearson correlation coefficients between log-transformed normalised intron usage ratios in introns identified across all conditions. Normalisation consists of regressing out experimental covariates similar to “Differential Splicing Analysis”, quantile normalisation and inverse-normal rank transformation. Only the 20,000 most variable introns across conditions were used.

Supplementary Figure 2: Level-2 REACTOME pathway enrichment analysis results in different conditions.

Level-2 REACTOME pathways enriched in differentially spliced genes across different stimulation conditions. Coloured labels on the left-hand side indicate the level-1 pathways that each pathway belongs to. After six hours, stimulation with CIL, LIL10 and sLPS captures all the level-2 pathways enriched across all six-hour conditions. After 24 hours, stimulation with PIC and IFN β captures all the level-2 pathways enriched across all 24-hour conditions. Double asterisks indicate significant enrichment (Bonferroni-corrected Enrichr P-value < 2.5x10^-3^ for 20 conditions) and single asterisks indicate nominal enrichment (Enrichr P-value < 0.05).

Supplementary Figure 3: Level-3 REACTOME pathway enrichment analysis results in different conditions.

Level-3 REACTOME pathways enriched in differentially spliced genes across different stimulation conditions. Coloured labels on the left-hand side indicate the level-1 pathways that each pathway belongs to. Double asterisks indicate significant enrichment (Bonferroni-corrected Enrichr P-value < 2.5x10^-3^ for 20 conditions) and single asterisks indicate nominal enrichment (Enrichr P-value < 0.05).

Supplementary Figure 4: Number of identified introns before and after quality control.

Number of introns identified in the intron clustering procedure of Leafcutter (before intron sQTL quality control; in red) and the number of introns that were used as input to map sQTLs (after intron sQTL quality control; in black). For quality control steps and parameters see Methods.

Supplementary Figure 5: Replication of effect sizes of lead SNP per intron between Macromap Ctrl_6/Ctrl_24 and naive macrophage sQTLs from Alasoo et al. 2018 and Nedelec et al. 2016.

MacroMap effect size (Ctrl_6 and Ctrl_24) are shown on the x-axes and effect sizes of datasets are shown on the y-axes. Both datasets were preprocessed as part of eQTL catalogue. ρ = Pearson’s correlation coefficient and π_1_=replication rate defined as 1- π_0_ in the qvalue package^79^.

Supplementary Figure 6: Distance between lead SNP and significant sQTL introns.

Distribution of the distance between the lead SNPs of significant sQTL effects (across all conditions) and transcription start site (TSS; in black) of the sQTL gene, 5’intron boundary (in blue) and 3’ intron boundary (in red).

Supplementary Figure 7: Colocalisation between eQTLs and sQTLs.

Proportion of significant sQTL effects that colocalise with an eQTL in the same gene and same condition (using a permissive PP4 threshold ≥ 0.1). Red line indicates the average proportion of sQTLs with sQTL/eQTL colocalisation ≥ 0.1 across conditions.

Supplementary Figure 8: Prevalence of immune disease colocalisations in MacroMap conditions.

Heatmap showing the number of high-confidence colocalisation events in each disease (PP4 ≥ 0.75), where there is colocalisation evidence in one condition and in more than one condition.

Supplementary Figure 9: Response colocalisations in each tested immune-mediated disease.

Proportion of high-confidence colocalisation events in each disease (PP4 ≥ 0.75) that are response sQTLs (LFSR < 0.05).

Supplementary Figure 10: Non-immune disease colocalisations show lower colocalisation yields compared to immune disease colocalisations.

Proportion of neuro-psychiatric disorder loci that colocalise with an eQTL, an sQTL or both (PP4 ≥ 0.75).

Supplementary Figure 11: Dependence of colocalisation yield on PP4 cutoff.

Percentage of loci that colocalise solely with an sQTL at PP4 threshold ≥ 0.75 and using different PP4 threshold for eQTL colocalisation (top). Percentage of loci that colocalise solely with an eQTL at PP4 threshold ≥ 0.75 and using different PP4 threshold for sQTL colocalisation (bottom).

Supplementary Figure 12: Per-condition colocalisation yields are similar across conditions.

Proportion of colocalised splice junctions in each condition coloured by intron usage ratio.

Supplementary Figure 13: Comparison of effect sizes between common- and low-usage splice junctions.

(Left) Cumulative distribution of absolute effect sizes for low-usage (black) and common-usage (orange) splice junctions that colocalise with disease-associated loci (Right) boxplot showing the difference in absolute effect sizes between common-usage and low-usage splice junction. Common- and low-usage splice junctions exhibit a small but significant difference in mean absolute effect sizes (mean absolute effect sizes=0.75 and 0.7, standard deviation=0.267 and 0.257, respectively; Kruskal-Wallis test P-value=8.9x10^-15^). Common- and low-usage splice junctions refer to splice junction with mean IUR > 0.1 and ≤ 0.1 in the high-usage genotype, respectively.

Supplementary Figure 14: Alignment scores of common- and low-usage introns

Distribution of mean alignment score in low-usage and common-usage splice junctions. Mean alignment score was calculated per sample and then the mean was calculated across all RNA-seq samples per condition for each colocalised splice-junction/condition pair. Common- and low-usage splice junctions refer to splice junction with mean IUR ≥ 0.1 and < 0.1 in the high-usage genotype, respectively.

Supplementary Figure 15: Multi-mapping ratios of common- and low-usage splice junctions.

Distribution of median multi-mapping ratio in low-usage and common-usage introns. Multi-mapping ratio was calculated per sample and then the median was calculated across all RNA-seq samples per condition for each colocalised intron/condition pair.

Supplementary Figure 16: Types of splice sites in common- and low-usage splice junctions.

Number of common-usage (top) and low-usage (bottom) splice junctions that feature canonical and non-canincal splice sites. Splice site information were obtained from the *.SJ.out.tab files that are output by STAR.

Supplementary Figure 17: Cumulative distribution of high-genotype IUR.

Cumulative distribution of high-genotype IUR for all post-QC introns (grey) and colocalised introns (red).

Supplementary Figure 18: Enrichment of low-usage introns in colocalised versus all post-QC tested introns at different high-genotype IUR cutoffs.

Enrichment test for low-usage colocalised introns based on different high-genotype IUR cutoffs for defining low-usage introns. Fisher’s exact test odds ratio for the enrichment of colocalised low-usage introns is shown on the y-axis and the high-genotype IUR used to define low-usage introns is shown on the x-axis. OR=Odds ratio. OR < 1 indicates depletion of low-usage introns among colocalised introns and OR > 1 indicates enrichment of low-usage introns among colocalised introns. Error bars represent 95% confidence intervals of Fisher’s exact test point estimate.

Supplementary Figure 19: Replication of low-usage introns in Intropolis.

Distribution of the number of Intropolis RNA-seq samples in which a colocalised low-usage intron can be detected in the Intronpolis database.


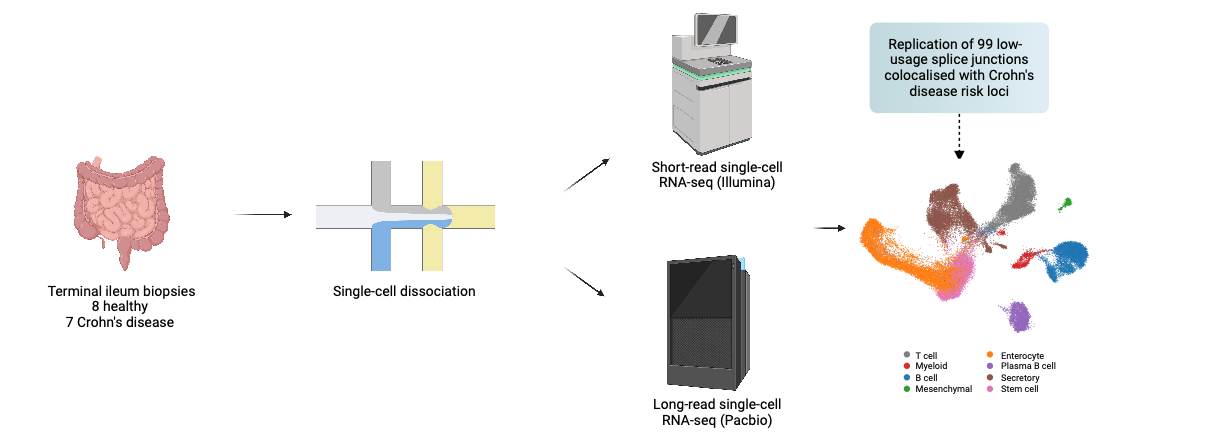


Supplementary Figure 20: Overview of long-read RNA-seq replication of low-usage introns.

Replication of 76 out of 99 low-usage splice junctions that colocalised with Crohn’s disease association signals in long-read RNA-seq data from 15 terminal ileum biopsies. UMAP based on gene-level counts shows cell types in the terminal ileum biopsies (including 1,203 myeloid cells). Diagram was created with BioRender.com.

Supplementary Figure 21: Replication of low-usage introns in long-read RNA-seq data.

Characteristics of the 76 splice junctions that colocalised with Crohn’s disease associated signals that were replicated in long-read RNA-seq data. (Left) Distribution of the total number of long reads covering the replicated splice junctions (log_10_ scale) (Middle) Distribution of the number of long-read samples where replication is detected (Right) Distribution of the number of cell barcodes where replication is detected.


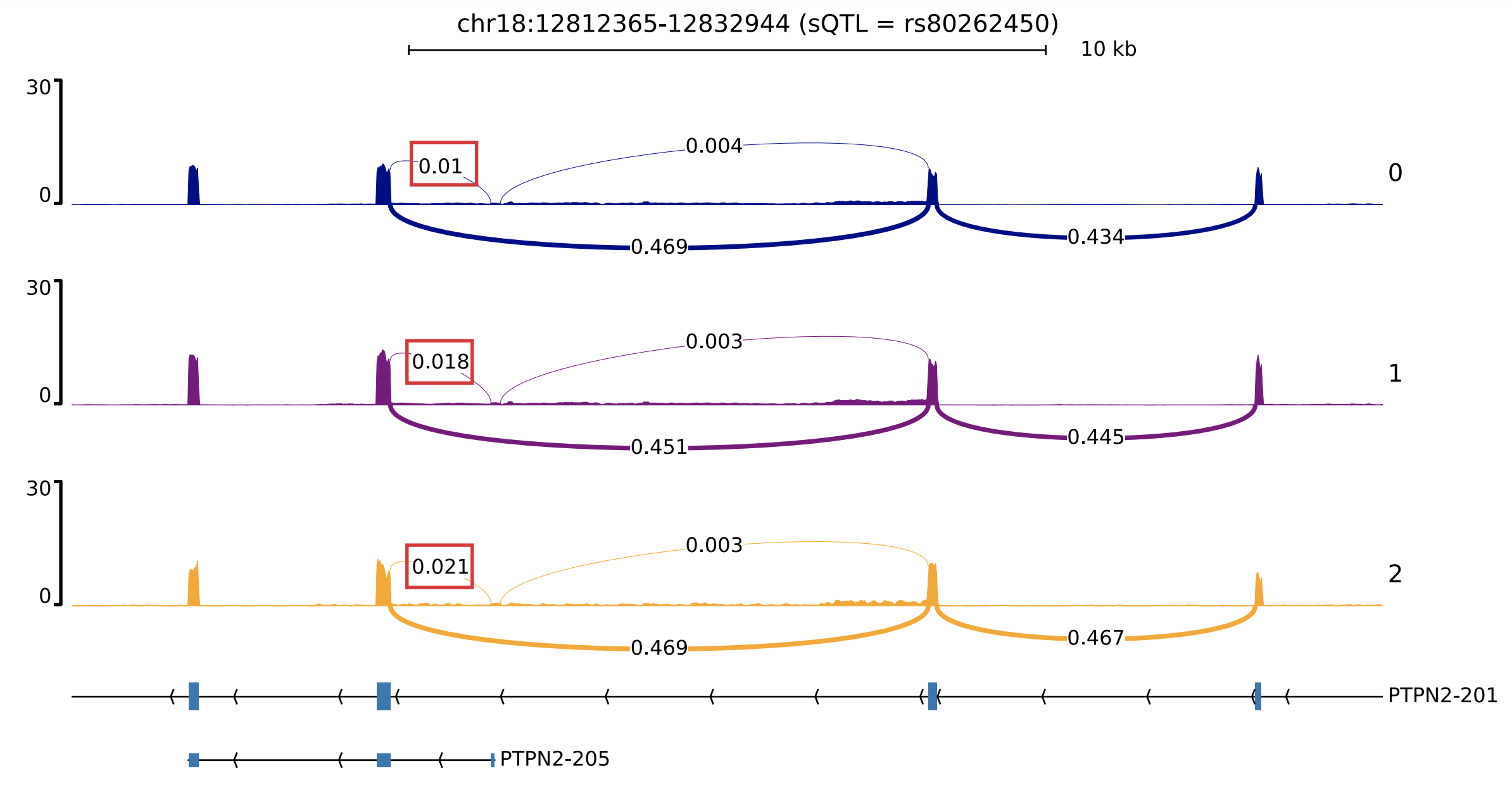


Supplementary Figure 22: RNA-seq coverage of the *PTPN2* intron cluster in Ctrl_6.

Bars represent the number of reads and arcs represent the usage of different introns, stratified by the number of copies of the minor allele (0,1 and 2). The splice junction that colocalises with the IBD risk locus in sLPS_6 is shown in a red box. Here, the sQTL effect size is smaller and therefore there is less variation with differing numbers of copies of the minor allele.

Supplementary Figure 23: GTEx colocalisation results in 18p11.21.

Heatmap of colocalisation PP4 values in all GTEx tissues in all genes in the IBD-assocaited locus 18p11.21. Colocalisation values with GTEx eQTLs are shown in the left heatmap and with GTEx sQTLs in the right heatmap. Colocalisation evidence exists for both a *PTPN2* sQTL and a *RP11-973H7.1* eQTL in multiple tissues including Colon_Transverse.


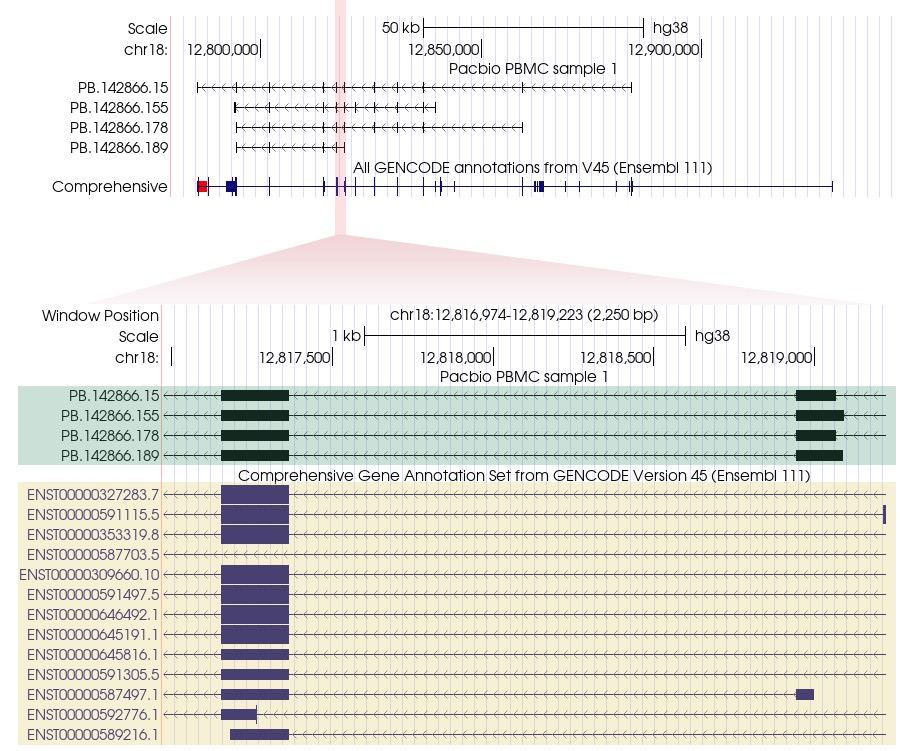


Supplementary Figure 24: *PTPN2* splice junction chr18:12817365-12818944 isoform diversity in a public long-read RNA-seq dataset.

Four *PTPN2* transcripts feature the splice junction chr18:12817365-12818944 in a Pacbio PBMC sample sequenced using long-read RNA-seq (highlighted in green; downloaded from downloads.pacbcloud.com/public/dataset/MAS-Seq/DATA-MAS-Revio-PBMC-1/). All *PTPN2* isoforms in GENCODE v45 are shown (highlighted in yellow).

Supplementary Figure 25: *DENND1B* RNA-seq coverage plot in CIL_24.

RNA-seq coverage of the intron cluster where the *DENND1B* sQTL effect is detected in CIL_24. Bars represent the number of reads and arcs represent the usage of different introns (the colocalised sQTL splice junction is indicated in a red box).


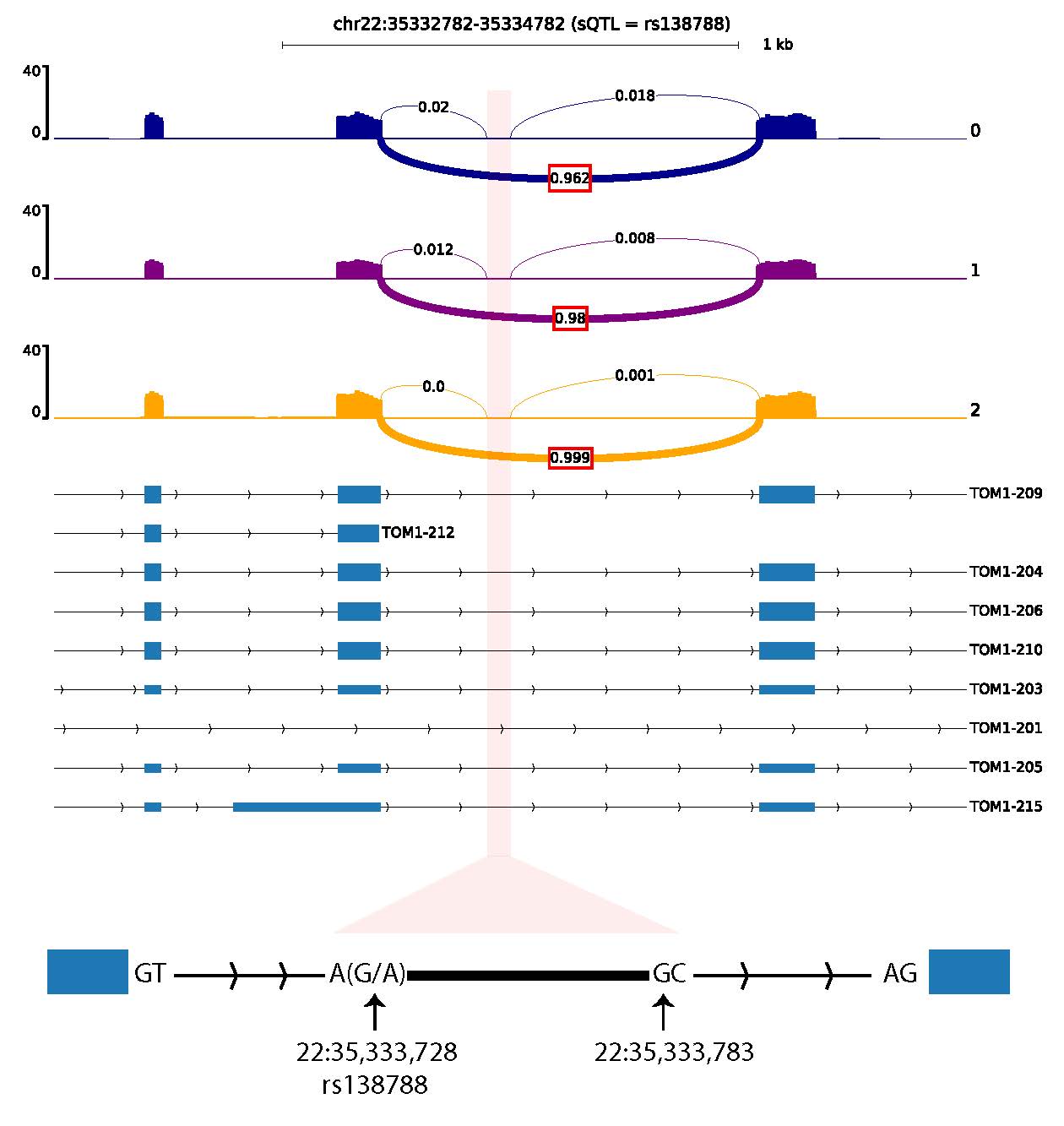


Supplementary Figure 26: *TOM1* RNA-seq coverage plot in CIL_6.

RNA-seq coverage of the intron cluster where the *TOM1* sQTL effect is detected in CIL_6 stratified by the number of copies of the minor allele (0, 1 and 2). Bars represent the number of reads and arcs represent the usage of different introns (the colocalised sQTL splice junction is indicated in a red box).

Supplementary Figure 27: IBD, *PTPN2* and *RP11-973H7.1* regional association plots.

GWAS regional association plots for IBD (top), and sQTL regional association plots for *PTPN2* (middle; PP4=0.995) and *RP11-973H7.1* eQTL (bottom; PP4=0.97) in Colon_Transverse in GTEx. Colors indicate LD with the lead IBD SNP rs80262450.

Supplementary Figure 28: Principal component correction in sQTL mapping.

Number of intron usage ratio principal components used as covariates (x-axis) versus the number of genes for which a significant sQTL effect was found (y-axis) coloured by time point (6=6 hours, 24=24 hours, D0=Day 0, D2=Day 2).
