# Supplementary material for: Splicing QTL mapping in stimulated macrophages associates low-usage splice junctions with immune-mediated disease risk
Source: Nat Commun. 2025 Aug 27;16:7205. doi: 10.1038/s41467-025-61669-2 (PMC12391537; doi:10.1038/s41467-025-61669-2)
Supplement: Supplementary file 2 — Description of Additional Supplementary Files [file 41467_2025_61669_MOESM2_ESM.pdf]

Description of Additional Supplementary Files

Supplementary Data 1: Concentrations of adjuvants used in each stimulation condition.

Supplementary Data 2: Experimental and epidemiological covariates for each HipSci cell line used.

Supplementary Data 3: Pathway enrichment analysis results for differential spliced genes (between each stimulation condition and control).

Supplementary Data 4: GWAS studies used for colocalisation analysis with both Macromap eQTL and sQTL.

Supplementary Data 5: Number of long-reads and number of cells after each step of long-read RNA-seq quality control.

Supplementary Data 6: Full statistical colocalisation results between the IBD locus 18p11.21 and MacroMap and GTEx sQTLs.

Supplementary Data 7: Full statistical colocalisation results between the IBD locus 22q12.3 and MacroMap and GTEx sQTLs.

Supplementary Data 8: Full statistical colocalisation results between the IBD locus 1q31.1 and MacroMap and GTEx sQTLs.

Supplementary Data 9: Numbers of RNA-seq samples for each stimulation condition.
